# Supraglottic p16+ squamous cell carcinoma during pregnancy: a case report and review of the literature

**DOI:** 10.1186/s40463-019-0378-z

**Published:** 2019-10-15

**Authors:** Jakob Pugi, Marc Levin, Michael Gupta

**Affiliations:** 1 Department of Surgery, Division of Otolaryngology-Head and Neck Surgery, McMaster University, Hamilton, ON Canada; 2 Michael G. DeGroote School of Medicine, McMaster University, 1280 Main St. West, Hamilton, ON L8S 4L8 Canada

**Keywords:** Supraglottic cancer, Cancer in pregnancy, Case report, Laryngeal cancer, Squamous cell carcinoma, Human papillomavirus

## Abstract

**Background:**

Head and neck cancer during pregnancy is uncommon. Specifically, laryngeal cancer in pregnancy has only been previously reported 10 times. HPV p16+ supraglottic cancer during pregnancy has never been described in the literature prior to this case report. This case is important to report to understand the most effective and safe diagnostic, treatment and follow-up options available for pregnant patients with laryngeal cancer.

**Case presentation:**

This report describes a case of a 33-year-old patient who was 24 weeks pregnant presenting with dysphonia and odynophagia. After laryngeal biopsy and MRI she was diagnosed with T3N1M0, stage three p16+ squamous cell carcinoma of the supraglottis. After inter-disciplinary consultation as well as extensive patient discussion, an awake tracheostomy, PEG tube placement and then elective C-section at 28 weeks’ gestation was completed. This was followed by chemoradiotherapy. The patient has remained free from disease with a healthy child at four years post-treatment.

**Conclusion:**

Supraglottic cancer during pregnancy is rare with only four previous cases reported in the literature. This case report elucidates the importance of including multiple specialities as well as patient preference in the decision-making process regarding treatment for patients with supraglottic cancer during pregnancy. Furthermore, diagnostic and treatment guidelines for pregnant patients with head and neck cancers should be established to promote the best possible oncological, obstetrical and neonatal care.

## Introduction

Supraglottic squamous cell carcinoma accounts for approximately one third of all laryngeal cancers [1]. The most common risk factors include smoking tobacco, alcohol consumption, asbestos exposure [2, 3] and oncoviruses such as the human papillomavirus (HPV) [4]. The large majority of patients, up to 97%, with laryngeal cancer are male in the fifth to seventh decade of life [1]. As such, laryngeal cancer diagnoses among young females are rare, with having this diagnosis during pregnancy being even more so.

**Table 1:**
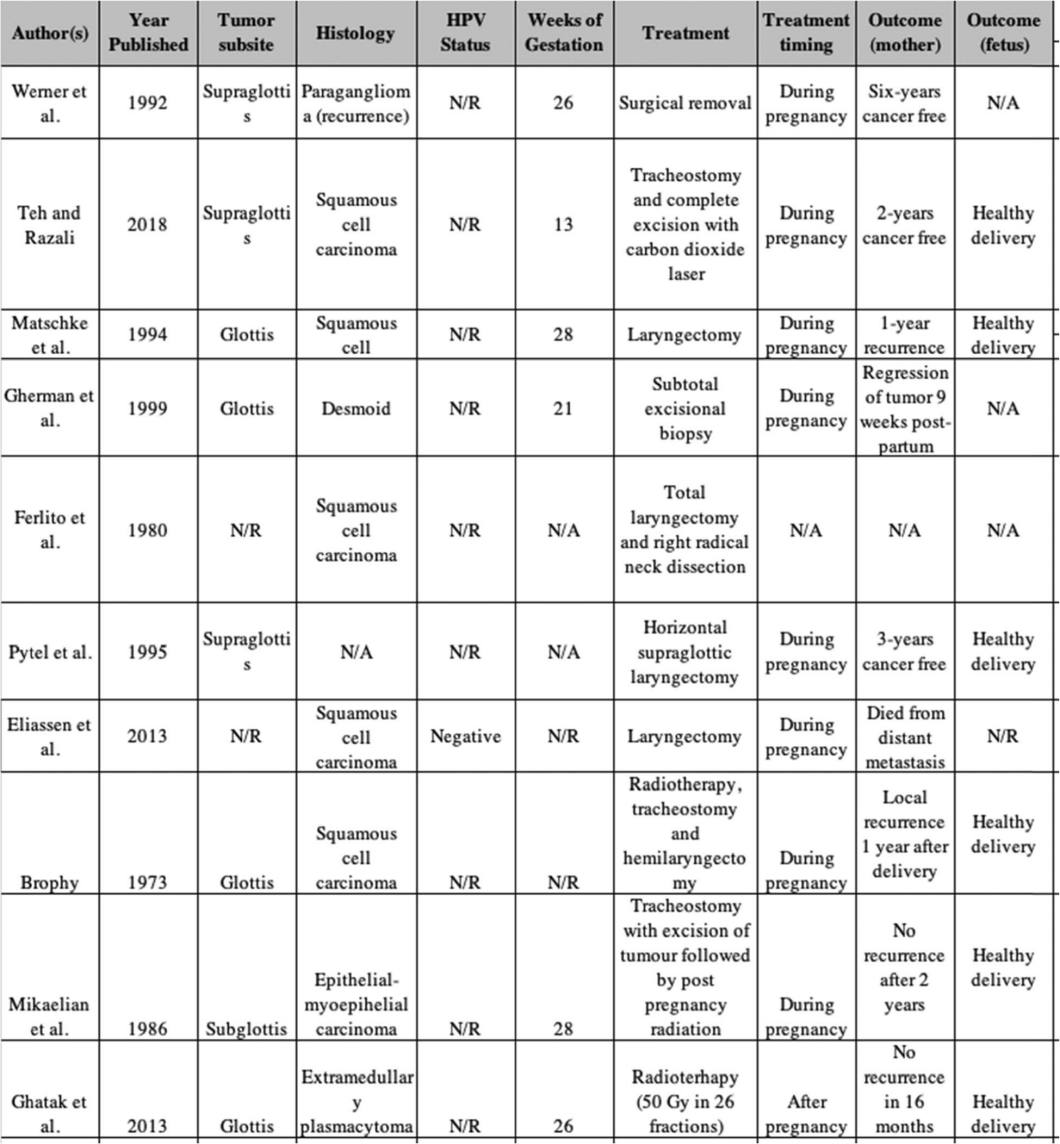
Laryngeal cancer during pregnancy literature review

N/R = not reported; N/A = not accessible

Head and neck cancer during pregnancy is complicated by unique medical, surgical and ethical dilemmas of selecting appropriate and effective treatment for the mother while minimizing harm to the fetus [5]. Many aspects of care have new implications to consider including imaging techniques, surgical considerations, and potential adjuvant treatments [5]. Timing of delivery versus instigation of treatment also becomes an intricate discussion with multiple care providers and the patient, with preferences of the patient playing a large and important role.

A number of case reports have been published on head and neck cancer treatment and outcomes during pregnancy [6–12]. Ten cases of laryngeal cancer during pregnancy exist in the literature [13–22], with one published case report specifically on supraglottic squamous cell carcinoma during pregnancy [14]. Other case reports on supraglottic cancer during pregnancy date back to the early 1990’s [13, 18]. There are no reports that described a p16+ supraglottic squamous cell carcinoma. Additionally, no articles have reviewed and summarized treatments and outcomes of laryngeal cancer during pregnancy. There have been many advances in precision chemotherapy and radiation therapy and increased use of interdisciplinary collaborative care have over the last two decades, which has changed the treatment options available for patient care.

We present a case report showcasing the utility of a combined surgical and chemoradiotherapy approach to a pregnant patient with supraglottic p16+ squamous cell carcinoma.

## Case report

A 33-year-old Caucasian female presented to our head and neck oncology clinic. She was 24 weeks pregnant at the time of presentation. She complained of a four-month history of dysphonia and odynophagia. Additionally, she noted shortness of breath on exertion as well as left-sided otalgia. She was otherwise healthy with a history of a tonsillectomy at age five, and a previous knee arthroscopy. She was taking prenatal vitamins, Tylenol and ranitidine. She had 10 pack year history and quit one week prior to initial consultation. She drank 5–7 alcoholic drinks per week prior to pregnancy and worked as a bartender.

Flexible fiberoptic nasolaryngoscopy revealed a lesion involving both vocal cords, fixing the left vocal cord with impaired mobility of the right vocal cord. The lesion extended from the cords to the ventricle and onto the false cords with partial involvement of the base of the epiglottis. Laryngeal biopsy revealed squamous cell carcinoma involve both the left and right supraglottis. In addition to the supraglottic lesion, magnetic resonance imaging (MRI) noted a 12.0 mm lymph node in the right neck level IIa. Hence, per the patient was diagnosed with T3N1M0, stage three p16+ squamous cell carcinoma of the supraglottis.

Extensive inter-disciplinary discussion was completed regarding this patient’s treatment plan. Experts from medical oncology, radiation oncology, obstetrics and gynecology, speech and language pathology and otolaryngology - head and neck surgery were included. Importantly, the patient’s perspectives and preferences were also included in the decision-making process. After much deliberation, it was decided to proceed with an awake tracheostomy, percutaneous endoscopy gastrostomy (PEG) tube placement and then elective C-section at 28 weeks’ gestation, followed by chemoradiotherapy (Cisplatinum 100 mg per m2 on days 1,22, and 43). The radiation dosage used was 7000 centigray in 35 fractions over 7 weeks. Treatment was successful and the patient has remained recurrence free with a healthy child at four years post-treatment. From a functional outcome perspective, the patient has stable dysphonia that has not improved over time. Additionally, she has a small glottic web superior to the vocal cords. Finally, the patient has no dysphagia or any other airway/breathing difficulties.

## Discussion

Cancer during pregnancy is uncommon, occurring in one out of every thousand pregnancies [23], making head and neck cancers during pregnancy an especially rare occurrence. Few case reports have been published describing cancers of oral, parotid, thyroid, tongue, palate, nasopharynx, maxillary sinus and supraglottic regions in pregnancy [5–12, 24], with laryngeal cancers being even less prevalent. Our extensive literature review revealed ten cases of laryngeal cancer [13–22] with only three of these being published within the last twenty years [14, 19, 22] (Table 1). More specifically, only one of these cases describes squamous cell carcinoma of the supraglottis with mention of HPV status [14]. The lack of evidence regarding HPV related supraglottic cancers in pregnant patients is expected given the reported rates of HPV infection in laryngeal cancer patients range from 0 to 27% [25–27]. Furthermore, only 30.5% of those patients with HPV related laryngeal cancers have supraglottic tumors [27].

The debate on which treatment modality is best for laryngeal cancer patients is constantly developing. A combination oncologic, quality of life and laryngeal function factors all contribute to the healthcare team and patient’s decision on treatment modality. Multiple possible treatments exist including radiation with or without chemotherapy, total laryngectomy, open partial laryngectomy or transoral laser microsurgery [28–32]. Despite these data, there are no recommendations available for treating laryngeal, specifically supraglottic cancer in pregnancy. Herein lies the challenge for the clinicians and surgeons in our case, in that chemotherapy and radiotherapy that would normally be given to a patient with a T3 N1 M0 supraglottic squamous cell carcinoma, would not be appropriate in a pregnant patient due to their associated maternal and fetal risks [33, 34]. In over 25% of cancers associated with pregnancies, all treatments are delayed until after delivery [34]. After consultation with our obstectrics and gynecology colleagues we agreed that providing this patient with chemoradiation therapy during pregnancy would pose potential unknown risks to the mother and fetus. Additionally, the patient did not want to undergo a total laryngectomy given her young age and length of surgery. It was also decided with the patient that having a laryngectomy at the same time as her first child would impact her ability to raise her child. Furthermore, our obstetrics and gynecology colleagues advised that induction of delivery of the fetus at 24 weeks gestational age would not be advised given the many associated risks with such extreme prematurity. In addition to these factors limiting our ability to treat this patient during pregnancy, our patient was having concerning airway symptoms such as dysphonia, odynophagia and dyspnea and required urgent airway management.

After consultation with anesthesiology, we decided to proceed with an awake tracheostomy to allow this patient a patent airway during her pregnancy and eliminate any possibility for airway obstruction. Safe and successful tracheostomies have been reported during pregnancy [35, 36]. With the confirmed patency of her airway following tracheostomy placement, we were confident we could wait one month (28 weeks gestation) until a viable and safe delivery would be possible. As such, the patient waited until 28 weeks’ gestation when she was induced and delivered a healthy child via Caesarean-section.

Management across the laryngeal cancer in pregnancy case reports found in our literature search were variable ranging from total laryngectomies during pregnancy to radiotherapy following delivery [22, 28]. In the case report of the other supraglottic squamous cell carcinoma a tracheostomy and complete excision with carbon dioxide laser was completed [14]. At our center, we recognized the dearth and inconsistency of evidence to guide our treatment of this patient. Hence, it was essential that we made our treatment decisions in an inter-disciplinary and patient-centered manner.

From a maternal outcome perspective, 60% of laryngeal cancer during pregnancy cases reported recurrence-free survival. The remaining cases unfortunately reported local recurrence and one case of distant metastasis causing patient death. All cases that reported on neonatal outcomes reported a healthy delivery and survival of the neonate following delivery. Hence in future cases of laryngeal cancer during pregnancy, mothers may be counselled about the successful neonatal survival in previous cases. They must however also be counselled on the risk for potential disease recurrence after treatment and delivery.

## Conclusion

Despite their rarity, head and neck cancer’s during pregnancy do occur. At our centre, we managed a patient with supraglottic p16+ squamous cell carcinoma during pregnancy. In the case of supraglottic cancer in pregnancy, the mother’s airway and oncological outcomes as well as the fetus’ viability for life are of the utmost priority. Once the neonate is delivered, the patient may then be able to undergo oncologic treatment for their cancer. Currently, there are no guidelines or consensus regarding the treatment of head and neck cancer’s during pregnancy. Head and neck cancers in this population reveal a unique challenge whereby oncologic, obstetric and anesthetic concerns must be considered. Hence, we urge for the collaborative creation of a treatment pathway for pregnant patients with head and neck cancers. With a unified understanding of treatment of head and neck cancer in this population, pregnant patients may be able to receive better oncological, obstetrical and neonatal care.

## Data Availability

Not applicable

## Abbreviations

HPV: Human papillomavirus
MRI: Magnetic resonance imaging
N/A: Not accessible
N/R: Not reported
PEG: Percutaneous endoscopy gastrostomy
